# Comparing Hydrogen Deuterium Exchange and Fast Photochemical Oxidation of Proteins: a Structural Characterisation of Wild-Type and ΔN6 β_2_-Microglobulin

**DOI:** 10.1007/s13361-018-2067-y

**Published:** 2018-09-28

**Authors:** Owen Cornwell, Sheena E. Radford, Alison E. Ashcroft, James R. Ault

**Affiliations:** Astbury Centre for Structural Molecular Biology, School of Molecular and Cellular Biology, Faculty of Biological Sciences, University of Leeds, Leeds, LS2 9JT UK

**Keywords:** HDX, FPOP, Amyloid, β_2_-microglobulin, Protein conformation, Protein dynamics

## Abstract

Hydrogen deuterium exchange (HDX) coupled to mass spectrometry (MS) is a well-established technique employed in the field of structural MS to probe the solvent accessibility, dynamics and hydrogen bonding of backbone amides in proteins. By contrast, fast photochemical oxidation of proteins (FPOP) uses hydroxyl radicals, liberated from the photolysis of hydrogen peroxide, to covalently label solvent accessible amino acid side chains on the microsecond-millisecond timescale. Here, we use these two techniques to study the structural and dynamical differences between the protein β_2_-microglobulin (β_2_m) and its amyloidogenic truncation variant, ΔN6. We show that HDX and FPOP highlight structural/dynamical differences in regions of the proteins, localised to the region surrounding the N-terminal truncation. Further, we demonstrate that, with carefully optimised LC-MS conditions, FPOP data can probe solvent accessibility at the sub-amino acid level, and that these data can be interpreted meaningfully to gain more detailed understanding of the local environment and orientation of the side chains in protein structures.

**Electronic supplementary material:**

The online version of this article (10.1007/s13361-018-2067-y) contains supplementary material, which is available to authorized users.

**Graphical Abstract:**
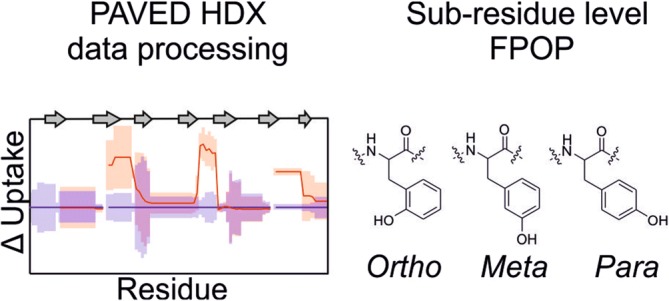
ᅟ

## Introduction

The study of protein structure and dynamics is essential to understand the nature of protein folding, function and mis-folding, the latter of which can result in aberrant protein aggregation associated with numerous amyloid diseases [1, 2]. Owing to its sensitivity and versatility, structural mass spectrometry (MS) is a powerful tool in this field and has been used to characterise individual components within heterogeneous mixtures [3–6].

**Figure 1:**
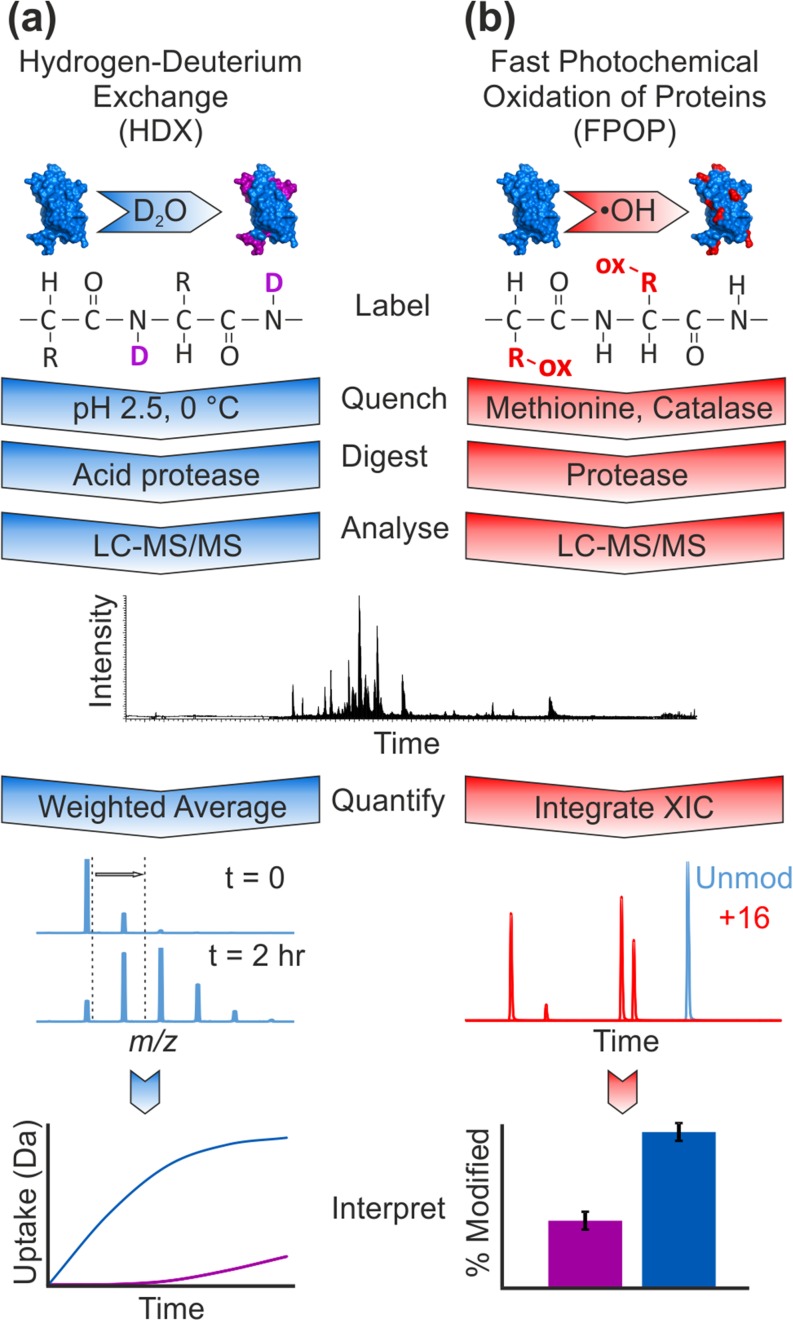
HDX-MS and FPOP-MS workflows. Structural interrogation of a protein can be carried out by labelling with either (**a**) deuterium (HDX) or (**b**) hydroxyl radicals (FPOP), followed by quenching (low temperature and low pH for HDX, or methionine and catalase for FPOP), and proteolysis. Separation and identification of the resulting peptides is achieved using LC-MS/MS. The extent of protein modification is determined from shifting isotope distributions (HDX) or integration of XICs (FPOP), which compare data from the unmodified peptides (cyan) with the modified peptides (red) between two states (blue and purple)

Two chemical labelling techniques of particular importance in the study of protein structure and dynamics are hydrogen deuterium exchange (HDX) [7, 8] and fast photochemical oxidation of proteins (FPOP) [9, 10]. Both are used frequently in conjunction with MS analyses. HDX is based on the principle of backbone amide proton exchange with deuterium. In a typical HDX-MS experiment (Figure 1a), the protein analyte is allowed to exchange protons with deuterium over a range of incubation times, before quenching the reaction at low temperature and low pH, followed by proteolytic digestion. Liquid chromatography-tandem MS (LC-MS/MS) is used to separate and identify the resulting peptides, where the mass increase from deuterium labelling is quantified as a weighted average from the peptide isotope distribution (Figure 1a). The mass increase over time allows the observation of changes in solvent accessibility and hydrogen bonding over long timescales (typically seconds to hours), which provides useful information on protein structure and dynamics [8].

HDX-MS is limited, however, by several inherent issues. Firstly, the reversible nature of deuterium labelling makes a certain degree of back-exchange after quenching inevitable, thus reducing the dynamic range of observable differences [11]. Similarly, the quench conditions necessary to reduce back-exchange are not suitable for many proteolytic enzymes, limiting HDX-MS experiments to acid proteases (almost exclusively pepsin, although other enzymes can be used) [12]. Secondly, it is not always possible to obtain residue level HDX-MS information due to the requisite use of non-ergodic peptide fragmentation techniques, such as electron transfer dissociation (ETD), and the careful tuning of MS conditions to reduce scrambling of the deuterium label during the analysis [13, 14].

By comparison with HDX-MS, FPOP is a less commonly used technique in which UV laser irradiation is employed to generate •OH radicals from H_2_O_2_ in solution (Figure 1b). These radicals react with solvent accessible amino acid side chains, especially large hydrophobic or sulphur-containing residues, resulting in a variety of side chain oxidations, most notably + 16 Da (+O), + 32 Da (+2O) and + 14 Da (CH_2_ to CO) mass additions [9, 15, 16]. The reaction is quenched with a reactive scavenger (e.g. methionine) before protease digestion and LC-MS/MS analysis is used to separate the oxidised peptides and identify modified residues. Typically, modifications are quantified by integration of the resulting extracted ion chromatograms (XICs) and are reported as a % modified of the total peptide ion count [17] (Figure 1b). This method offers a probe of solvent accessibility that labels side chains irreversibly, so no back-exchange of the label is possible, and thus lengthy analytical procedures can be employed to gain maximum information from the resulting data.

FPOP has other characteristics that make it a useful complementary technique to HDX. Firstly, quenching the reaction can be achieved at neutral pH and room temperature [17, 18] and hence a wide range of proteases, or combinations of proteases, can be used to digest the protein. Secondly, FPOP labels side chains on a microsecond-millisecond timescale [9, 19, 20], which are faster labelling times than those typically accessible to HDX-MS experimental setups. In principle, the covalent and residue-specific nature of the FPOP label mean that traditional MS/MS ergodic fragmentation techniques, e.g. collision-induced dissociation (CID), can be used to identify the oxidised side chain to residue level resolution [17, 21–23]. Like HDX, the side chain of the residue in an FPOP experiment affects its propensity to label [16]. However, compared with the wealth of knowledge available concerning the effect of primary amino acid sequence and solvent conditions on deuterium exchange [24], the effect of nearby side chains and solution conditions on FPOP labelling are not well understood.

Recently, FPOP and HDX have been used in several studies as complementary methods to study epitope mapping and higher order structure [22, 23, 25, 26]. However, given that FPOP primarily labels hydrophobic residues, we sought here to explore the usefulness of FPOP in the study of aggregation-prone proteins, where surface exposure of hydrophobic side chains is often involved in the unwanted self-polymerisation process [2, 27]. HDX-MS has already proved valuable in this field [27]. Consequently, we decided to use the well-characterised protein β_2_-microglobulin (β_2_m), and it is more amyloidogenic variant, ΔN6, as a model system with which to compare the utility of HDX and FPOP to reveal differences in the dynamical properties of these proteins that may be linked to their different propensities to form amyloid.

**Figure 2:**
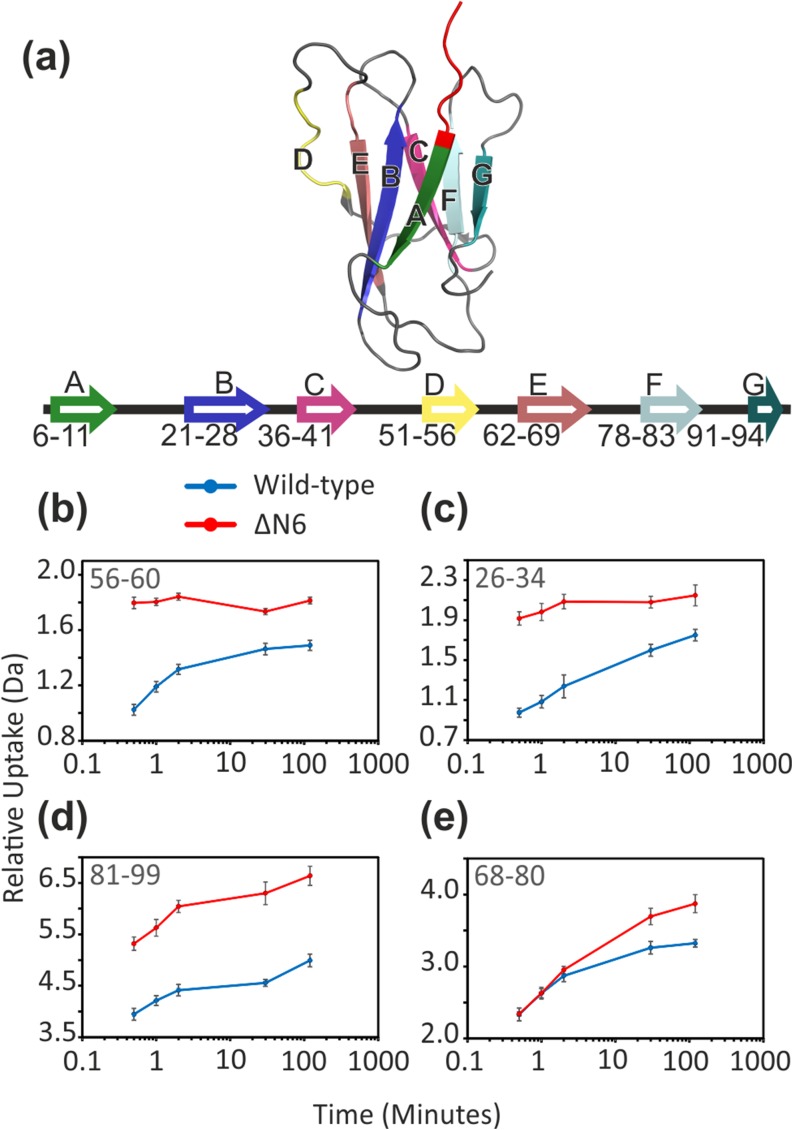
(**a**) Ribbon diagram showing the structure of wild-type human β_2_m (PDB: 2XKS [29]). The sequence positions of the seven β-strands are shown below in the same colour scheme. The six N-terminal residues, absent in ΔN6, are shown in red. (**b**–**e**) HDX uptake plots of wild-type (blue) and ΔN6 (red) peptides in four different regions: (**b**) residues 56–60, D-E loop; (**c**) residues 26–34, B strand and B-C loop; (**d**) residues 81–99, C-terminus; and (**e**) residues 68–80, E-F loop. Error bars show standard deviation (*n* = 5). Residues covered by each peptide (including each N-terminal residue) are shown in the top left corner of each plot

Recombinant human β_2_m is a 100-residue protein (11,860.4 Da) that forms the non-covalently bound light chain of the class I major histocompatibility complex [28]. Native β_2_m has a seven-stranded β-sandwich structure, in which strands A, B, D, and E form one β-sheet, and strands C, F, and G form the second β-sheet (Figure 2a) [30, 31]. The two β-sheets are tethered by a single disulphide bond linking Cys25 and Cys80 in strands B and F, respectively [32]. Aggregation of primarily full-length, wild-type β_2_m to form classical amyloid fibrils occurs in patients who have been subjected to long-term haemodialysis, resulting in the disorder dialysis-related amyloidosis [33]. However, in addition to the intact wild-type protein, up to 30% of β_2_m extracted from fibrils ex vivo contains the amyloidogenic truncation variant of the wild-type protein lacking the six N-terminal amino acids, ΔN6 [34, 35].

ΔN6 is considered a structural mimic of the amyloidogenic precursor of β_2_m amyloidosis, containing a *trans* His-Pro peptide bond at position 32 instead of the native *cis* isoform [29, 36]. The structural dynamics of wild-type and ΔN6 β_2_m have been studied previously by NMR relaxation methods [29, 37], HDX-NMR [38, 39], HDX-MS [34, 40–42] and various other biophysical techniques, but the mechanism by which truncation of the N-terminal hexapeptide enhances amyloidogenicity remains unclear.

Here, we characterise wild-type and ΔN6 β_2_m by peptide level HDX-MS, presenting our data using a novel visualisation and processing algorithm developed in-house, and show that our results are consistent with previous studies on β_2_m dynamics. We then compare these data with residue level FPOP labelling for both proteins and show how FPOP complements the HDX data with information on side chain solvent accessibility. Further, we demonstrate that with optimised LC separation and MS settings, FPOP-LC-MS/MS can be used to quantify modifications at the sub-amino acid level, and that these data can be interpreted meaningfully to attain higher resolution information on protein structure and dynamics.

## Methods

### Protein Preparation

Protein samples were expressed recombinantly as described previously [43].

### FPOP Followed by LC-MS/MS

The FPOP experimental set-up used was as described previously [17, 18]. Immediately prior to laser irradiation, 1 μl 5% *v/v* H_2_O_2_ was added to 100 μl of protein (10 μM) (final H_2_O_2_ concentration 0.05% *v/v* H_2_O_2_), containing 20 mM L-histidine in 10 mM potassium phosphate buffer pH 7.4. The capillary outflow following laser irradiation was collected in an Eppendorf tube containing 20 μl quench solution (100 mM L-methionine, 1 μM catalase in 10 mM potassium phosphate buffer pH 7.4) and placed immediately on ice.

The single disulphide bond in irradiated protein samples was reduced (incubation with 10 mM dithiothreitol (DTT) for 1 h, 55 °C at 500 rpm), and the resulting thiols alkylated (incubation in the dark with 55 mM iodoacetamide for 45 min, 20 °C at 500 rpm) and digested with chymotrypsin (1:50 *w/w* ratio of enzyme to protein, incubated at 37 °C for 18 h at 500 rpm).

The resulting chymotryptic peptides (1 μl at 0.5 μM peptide concentration) were injected onto an ultra performance liquid chromatography (UPLC) M-Class Acquity system equipped with a C18 column (75 μm × 150 mm, Waters Ltd., Wilmslow, Manchester, UK) and separated by gradient elution of 1–50% MeCN (0.1% *v/v* formic acid) in H_2_O (0.1% *v/v* formic acid) over 60 min at 0.3 μl min^−1^.

Peptides were analysed using a Q Exactive Plus Orbitrap mass spectrometer (ThermoFisher, Bremen, Germany) in data-dependent acquisition mode. The top five most intense ions were selected for high energy HCD fragmentation. The maximum injection time for MS^2^ acquisition was set to 300 ms, and dynamic exclusion was reduced from 30 to 3 s to allow fragmentation of isobaric FPOP modifications that elute close together.

Peptides were identified using PEAKS 8.5 software (Bioinformatics Solutions Inc., Waterloo, ON, Canada). Mass additions of + 16, + 32 and + 14 Da were searched to identify modified peptides. Residue level quantification of FPOP modifications was achieved using Xcalibur software (v4.0.27.19; ThermoFisher, Bremen, Germany) by integration of modified and unmodified peaks generated from extracted ion chromatograms using the following equation:

1 $$ \% modified=\frac{\sum {modified}_{Peak}}{unmodified+\sum {modified}_{All\  peaks}} $$

XICs were generated by extracting the *m*/*z* of the base peak of each peptide isotope distribution, for each observed charge state.

### HDX Followed by LC-MS/MS

HDX-MS experiments were carried out using an automated HDX robot (LEAP Technologies, Fort Lauderdale, FL, USA) coupled to an M-Class Acquity LC and HDX manager (Waters Ltd., Wilmslow, Manchester, UK). Thirty microlitres of protein solution containing 8 μM of either wild-type or ΔN6 β_2_m in equilibration buffer (10 mM potassium phosphate buffer pH 7.4) was added to 135 μl deuterated buffer (10 mM potassium phosphate buffer pD 7.4) and incubated at 4 °C for 30, 60, 120, 1800 or 7200 s. Following the labelling reaction, samples were quenched by adding 50 μl of the labelled solution to 100 μl quench buffer (10 mM potassium phosphate, 2 M guanidine-HCl, 200 mM tris(2-carboxyethyl)phosphine pH 2.2) giving a final quench pH ~ 2.5. Fifty microlitres of quenched sample (ca 24 pmol) was passed through an immobilised ethylene-bridged hybrid (BEH) pepsin column (Waters Ltd., Wilmslow, Manchester, UK) at 500 μl min^−1^ (20 °C) and a VanGuard Pre-column Acquity UPLC BEH C18 (1.7 μm, 2.1 mm × 5 mm, Waters Ltd., Wilmslow, Manchester, UK) for 3 min. The resulting peptic peptides were transferred to a C18 column (75 μm × 150 mm, Waters Ltd., Wilmslow, Manchester, UK) and separated by gradient elution of 0–40% MeCN (0.1% *v/v* formic acid) in H_2_O (0.3% *v/v* formic acid) over 7 min at 40 μl min^−1^.

The HDX system was interfaced to a Synapt G2Si mass spectrometer (Waters Ltd., Wilmslow, Manchester, UK). HDMS^E^ and dynamic range extension modes (data independent analysis (DIA) coupled with IMS separation) were used to separate peptides prior to CID fragmentation in the transfer cell [44].

HDX data were analysed using PLGS (v3.0.2) and DynamX (v3.0.0) software supplied with the mass spectrometer. Restrictions for identified peptides in DynamX were as follows: minimum intensity 1000, minimum products per amino acid 0.3, max sequence length 25, max ppm error 5, file threshold 4/5.

To visualise data and generate difference plots, data were processed using an in-house developed algorithm (named PAVED) written in R and Java. This approach combines the mean relative fractional uptake values (and their standard deviations) per residue, per time-point, for all peptides covering a given residue (subtracting the N-terminal residue due to back-exchange) using the following two equations:

2 $$ {\overline{X}}_{\mathrm{C}}=\frac{\sum_{i=1}^z{n}_i{\overline{X}}_i}{\sum_{i=1}^z{n}_i} $$

3 $$ {S_{\mathrm{c}}}^2=\frac{\sum_{i=1}^z{n}_i\left[{S_i}^2+{\left({\overline{X}}_i-{\overline{X}}_C\right)}^2\right]}{\sum_{i=1}^z{n}_i} $$

where ${\overline{X}}_{\mathrm{C}}$ is the combined mean relative fractional uptake for a given residue at a given time-point, *n*_*i*_ is the number of replicates for peptide *i*, ${\overline{X}}_i$ is the mean relative fractional uptake for peptide *i* at a given time-point, *S*_c_ is the combined standard deviation for a given residue at a given time-point and *S*_*i*_ is the standard deviation of peptide *i* at a given time-point. Peptides *i* to *z* are all the peptides that cover an amino acid position, excluding the N-terminal residue of each.

Statistical analysis of the combined means and standard deviations was performed using one way ANOVA followed by post hoc Tukey tests [45].

## Results and Discussion

### Analysis of β_2_m and ΔN6 Dynamics Using HDX LC-MS/MS

Peptide level HDX-MS analyses of wild-type and ΔN6 β_2_m were first carried out to determine differences in deuterium uptake introduced as a result of the truncation of the N-terminal six amino acids in ΔN6 and to enable comparison of the HDX results with those of FPOP. The proteins were dissolved in 10 mM sodium phosphate buffer and HDX carried out for different lengths of time at 4 °C, pH 7.4 (see Methods). Previous results have shown that the proteins undergo complete HDX within that time at higher temperatures [29, 34, 37–42].

Following HDX, on-line pepsin digestion of both wild-type and ΔN6 β_2_m yielded a total of 46 peptides that passed the peptide identification restrictions in both proteins (see Methods), covering ~ 95% of the sequence (Supp. Info. Fig. S1). Peptides identified in only one of the two proteins were not used since for these peptides, a direct comparison of the behaviour of the two proteins could not be made. The N-terminal amino acid of each peptide was not included in the analysis due to unpreventable back-exchange, leaving just two internal residues not covered by any peptide: Tyr-26 and Arg-81. Several peptides showed increased deuterium uptake in ΔN6 relative to the wild-type protein which was apparent after only 30 s of deuterium labelling (Figure 2b–d). Peptide 56–60 (Figure 2b) shows deuterium uptake in ΔN6 reaching a plateau at the earliest labelling time-point (30 s), whereas the wild-type protein had yet to reach the equivalent deuterium uptake after 2 h. Similarly, peptide 26–34 arising from ΔN6 (Figure 2c) reaches a plateau after 2 min incubation with deuterium, while the respective wild-type peptide continues to increase in mass for the duration of the labelling experiment.

Multiple overlapping peptides covering these regions were also identified (Supp. Info. Fig. S2). However, the overlapping nature of the peptides complicates analysis, and makes combining and visualising the data to determine the regions of most significant difference challenging. The DynamX software, used here to curate and assign the exchange data, generates butterfly plots which show uptake differences as a function of peptide rather than amino acid position, and mass rather than relative fractional uptake. These properties can make it difficult to visualise the regions of proteins that undergo differential exchange.

To overcome these difficulties in data presentation, an in-house processing algorithm was developed to take the comma-separated value (csv) output files from the DynamX program and, drawing on previously published processing methods, visualise the data [27, 46]. Named PAVED (positional averaging for visualising exchange data), this process uses the deuterium uptake and standard deviation of deuterium uptake per time-point for each peptide covering an amino acid position in order to determine a combined mean and standard deviation for each position (see Methods, Eqs. 2 and 3). Using ANOVA and post hoc Tukey statistical tests, these combined mean uptake values are then compared in order to determine significant differences in uptake between the different proteins. The advantage of this simple approach is that it consistently uses all the data available from all the peptides detected in an experiment to give a readily interpretable visualisation of the uptake differences identified. No curve fitting is required, and this method avoids the complication of a ‘subtraction analysis’ of overlapped peptides [47]. ANOVA and post hoc Tukey tests are used so that multiple states can be compared on the same plot if required. It should be emphasised, however, that this tool is strictly for visualisation purposes only, the calculations outlined above are not meant to be taken as residue level uptake measurements.

**Figure 3:**
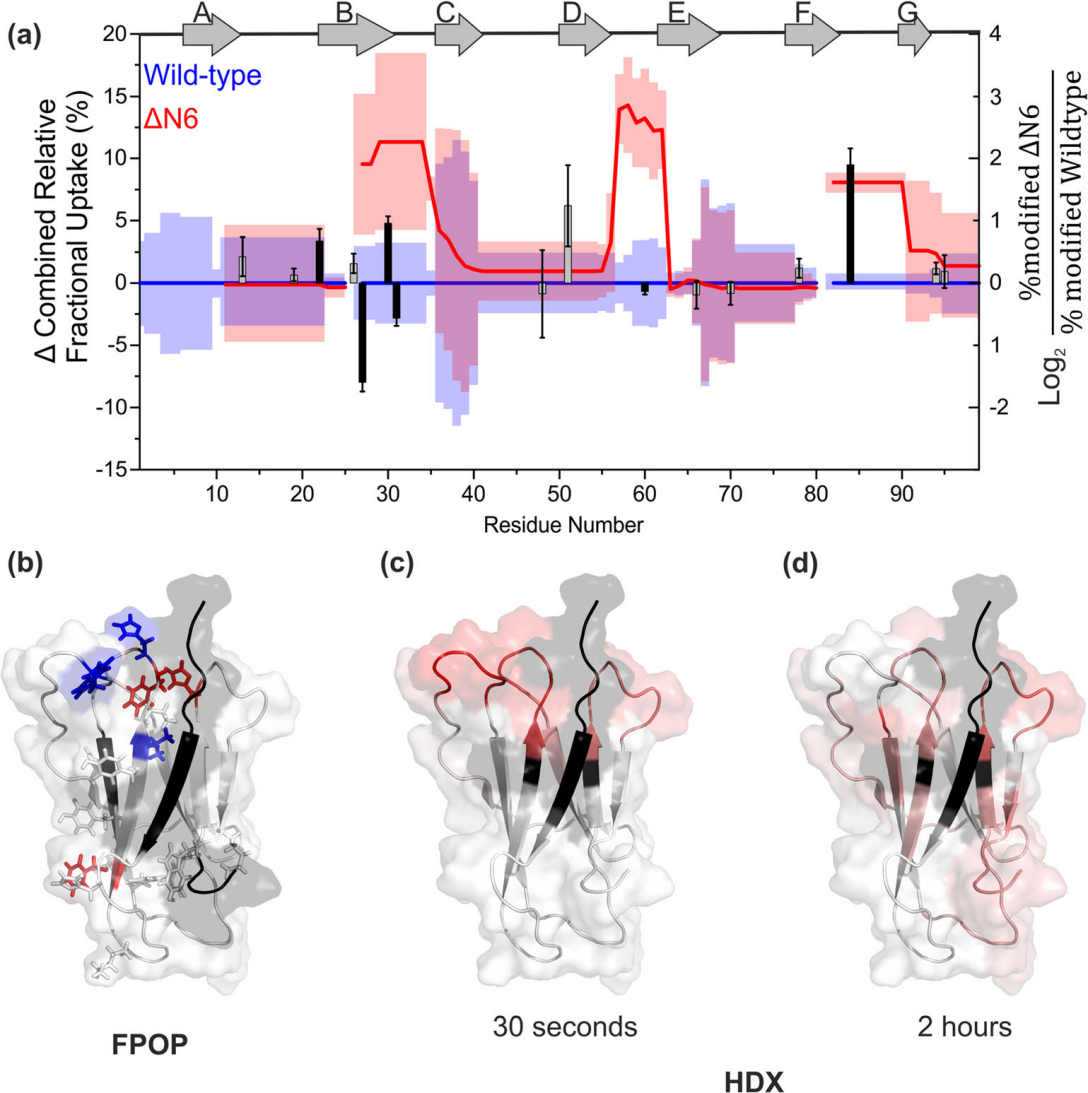
Differences in main chain dynamics between wild-type and ΔN6 β_2_m highlighted by use of HDX and FPOP, followed by proteolysis and LC-MS/MS analysis. (**a**) HDX difference plot using PAVED showing difference in combined relative fractional uptake after 30-s deuterium labelling (left y-axis) between ΔN6 (red line) and wild-type β_2_m (blue line set to zero for reference). Shaded regions (red = ΔN6, blue = wild-type) show combined standard deviation per residue. A bar chart of the FPOP data has been overlaid on the HDX difference plot showing residues modified by FPOP as a ratio of %modified ΔN6/%modified wild-type β_2_m plotted on a Log_2_ scale to illustrate fold change (right y-axis). Bars have been shaded to illustrate statistically significant differences (*p* < 0.05, solid black) or no significant change (grey). Error bars for FPOP show relative standard deviation, *n* = 3. (**b**) Side chains of residues modified by FPOP mapped onto the structure of β_2_m. No significant difference = grey, more labelling in wild-type = blue, more labelling in ΔN6 = red. (**c**, **d**) Statistically significant relative fractional uptake differences in HDX (*p* < 0.05 based on combined mean and combined standard deviation per residue) are shown (**c**: *t* = 30 s) and (**d**: *t* = 2 h). Red regions indicate significantly higher deuterium uptake in ΔN6 relative to the wild-type protein. In (**b**), (**c**), and (**d**), missing residues and the N-terminal truncation are shown in black. PDB: 2XKS [29]

Figure 3a shows the difference plot generated by PAVED for the 30 s labelling time-point comparing wild-type and ΔN6 β_2_m. The significant differences in deuterium uptake between the two proteins at this time-point are localised to the B strand (residues 21–28) and the B-C, D-E and F-G loops (residues 29–35, 57–61 and 84–90, respectively). Structurally, these regions of difference are directly proximal in 3D visualisation to the N-terminal truncation present in ΔN6 (Figures 2a, 3c) and highlight significant changes in the protein’s dynamics resulting from the removal of the N-terminal six amino acid residues, consistent with previous reports [29, 37].

Longer HDX incubation times (30 min and 2 h) begin to show increased deuterium uptake in ΔN6 in the E-F loop (Figure 2e) and the C-terminus, with other small increases (< 5% difference in relative fractional uptake) throughout the length of the protein (Figure 3d; Supp. Info. Fig. S3). This is consistent with previous intact HDX-MS data [34] and the known difference in stability between the two proteins [36].

### Analysis of β_2_m and ΔN6 Dynamics Using FPOP LC-MS/MS

Having identified several regions that show differences in dynamic behaviour in wild-type and ΔN6 β_2_m by peptide level HDX, we next sought to compare these data with results derived from the FPOP labelling method, so that structural and dynamical differences between the two proteins could be evaluated directly at the residue level. After independent oxidative labelling of each protein at pH 7.4 with hydroxyl radicals, wild-type and ΔN6 β_2_m were each subjected to proteolysis with chymotrypsin and the resulting mixtures of peptides in each case were analysed using LC-MS/MS. Seven chymotryptic peptides, covering 88% of the ΔN6 sequence, were found to be present reproducibly in the digests of both proteins (Supp. Info. Fig. S4) and were subsequently used for FPOP quantification.

By examining the MS/MS spectra and XICs of each peptide, we identified 17 different residues as modification sites (Table 1), indicating that approximately 20% of side chains in the ΔN6 sequence had been labelled successfully. This labelling coverage is consistent with previous FPOP experiments for other proteins [48]. The same residues were found to be modified in both the wild-type and ΔN6 β_2_m with the exception of Ile35 which, in the wild-type protein, was 0.05 ± 0.01% modified but was not observed to oxidise in ΔN6. Analysis of the NMR structures of the wild-type protein and ΔN6 [29] show a twofold lower solvent accessible surface area (SASA) of this residue in the truncation variant. This change, coupled with the low reactivity of Ile to hydroxyl radicals, is likely responsible for the absence of a modified Ile35 peptide in ΔN6. Two thirds of the side chains that labelled are in the top six residue types that are most reactive to hydroxyl radicals (Cys, Trp, Tyr, Met, Phe and His) [16]. Interestingly, Phe56 and Phe62 were not observed to label in either protein, despite the high reactivity of phenylalanine with hydroxyl radicals, and the high solvent accessibility of these residues in the NMR structures of both proteins [29]. Given the proximity of these residues to the solvent exposed Trp60 side chain, a residue significantly more reactive than phenylalanine with hydroxyl radicals, it is reasonable to conclude that the hydroxyl radicals nearby would preferentially label this side chain, leaving both Phe56 and Phe62 unlabelled. Similar sequence effects in FPOP have been observed previously in protein unfolding experiments [49]. Two adjacent peaks, both in the XIC of the + 16 Da modified peptide 88–95 (Supp. Info. Fig. S5), were left unassigned as, in both cases, MS/MS spectra were only sufficient to narrow the modification site to either Pro90 or Lys91. Neither of these modified peptides showed a significant difference in the % modified between the wild-type protein and ΔN6.

**Table 1:** Residues in Wild-Type and ΔN6 β_2_m Labelled by Use of FPOP. Residue Types Are Ordered According to Their Reactivities in Hydroxyl Radical Labelling from Highest (Trp) to Lowest Reactivity (Lys). Detailed Assignment Data for All Residues Can Be Found in Supp. Info. Table S1

Residues modified by FPOP	
Residue type	Position
Tryptophan	60, 95
Tyrosine	26, 67, 78
Phenylalanine	22, 30, 70
Histidine	13, 31, 51, 84
Isoleucine	35 (wild-type only)
Valine	27
Lysine	19, 48, 94

**Figure 4:**
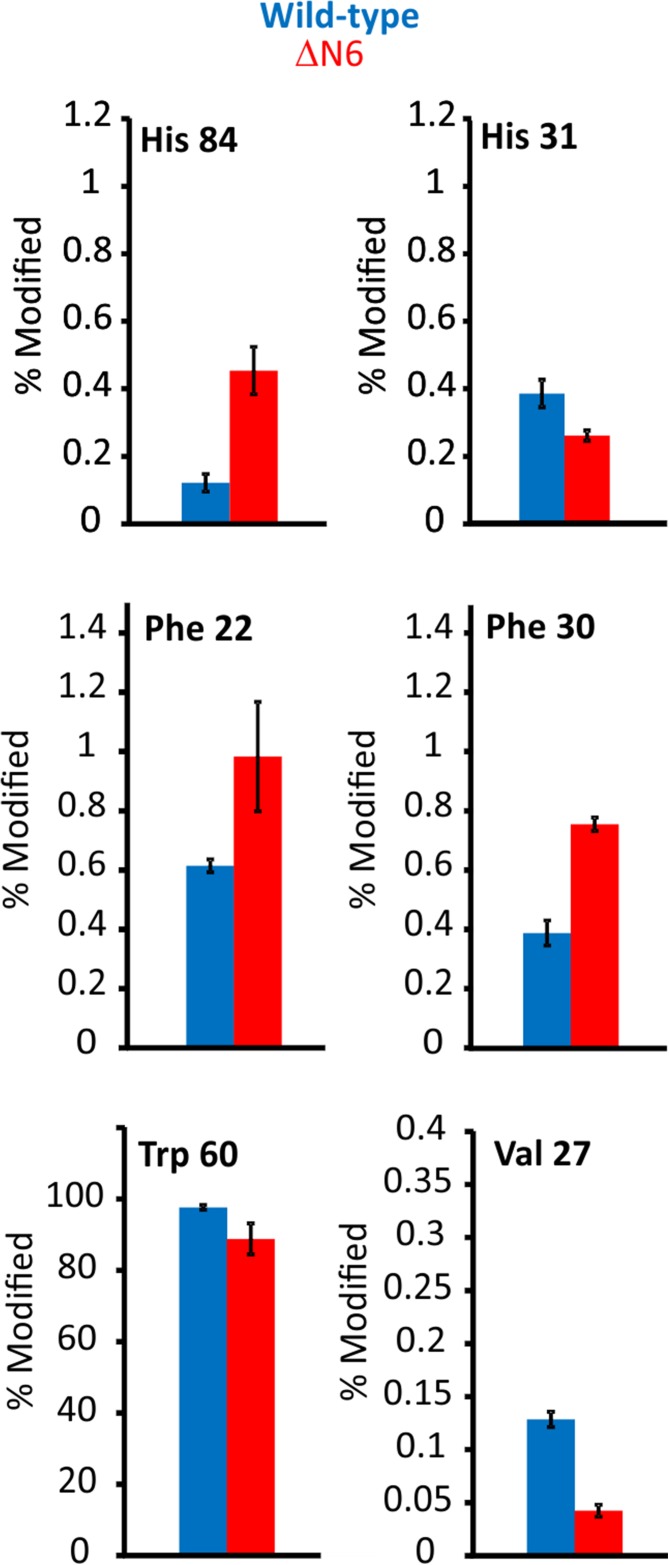
Residue level quantification of six FPOP modifications in wild-type (blue) and ΔN6 (red) β_2_m that showed statistically significant differences between the two proteins (*p* < 0.05). Val27, His31 and Trp60 show the wild-type protein to undergo more labelling while Phe22, Phe30 and His84 show ΔN6 to be labelled more. Error bars show standard deviation; *n* = 3

After XIC quantification, six of the 17 modified residues showed significantly different degrees of modification between the two proteins (*p* < 0.05): Phe22, Val27, Phe30, His31, Trp60 and His84 (Figure 4). Interestingly, with the exception of Phe22, these residues are within regions which indicated significant structural differences when subjected to HDX at the shorter incubation times (Figure 3a), and are similarly localised to the region proximal to the N-terminal truncation (Figure 3b). Interestingly, the more disperse differences shown by HDX after 2-h deuterium labelling highlight a time dependence of the agreement between the two techniques, where shorter HDX labelling times show more agreement with the FPOP data than longer incubation periods, likely resulting from the increased sensitivity of short timescale HDX to solvent access. Whereas the HDX data showed no regions of the wild-type protein that labelled more than the truncation variant, FPOP quantification shows specific residues with both significantly higher, and significantly lower, labelling in ΔN6 compared with the wild-type protein. Together, these data highlight the complementary nature of FPOP and HDX suggested by previous reports [22, 26]. This is demonstrated here by the observation that FPOP labelling and HDX labelling at the shortest incubation times both indicate differences localised to the region surrounding the N-terminal truncation.

### Analysis of FPOP Structural Isomers

**Figure 5:**
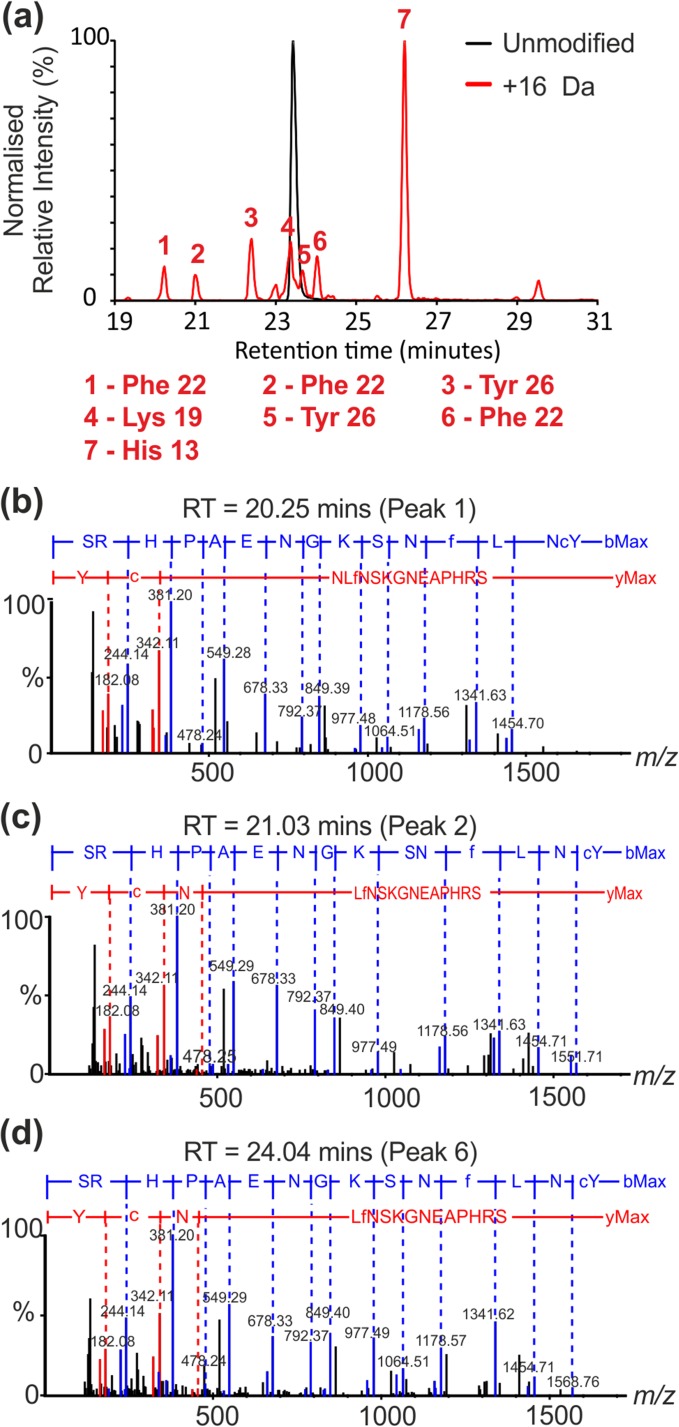
FPOP modifications on peptide residues 11–26 (sequence: SRHPAENGKSNFLNCY). (**a**) Overlaid extracted ion chromatograms of the unmodified (black peak; *m*/*z* 631.95, *z* = 3) and + 16 Da modified (red peaks; *m*/*z* 637.28, *z* = 3) peptides. (**b**–**d**) Phe22 (peaks 1, 2 and 6) was identified as the modified residue by MS/MS at three different retention times (**b**) 20.25, (**c**) 21.03, and (**d**) 24.04 min, respectively. Y-series ions are denoted in red and b-series ions in blue

**Scheme 1:**
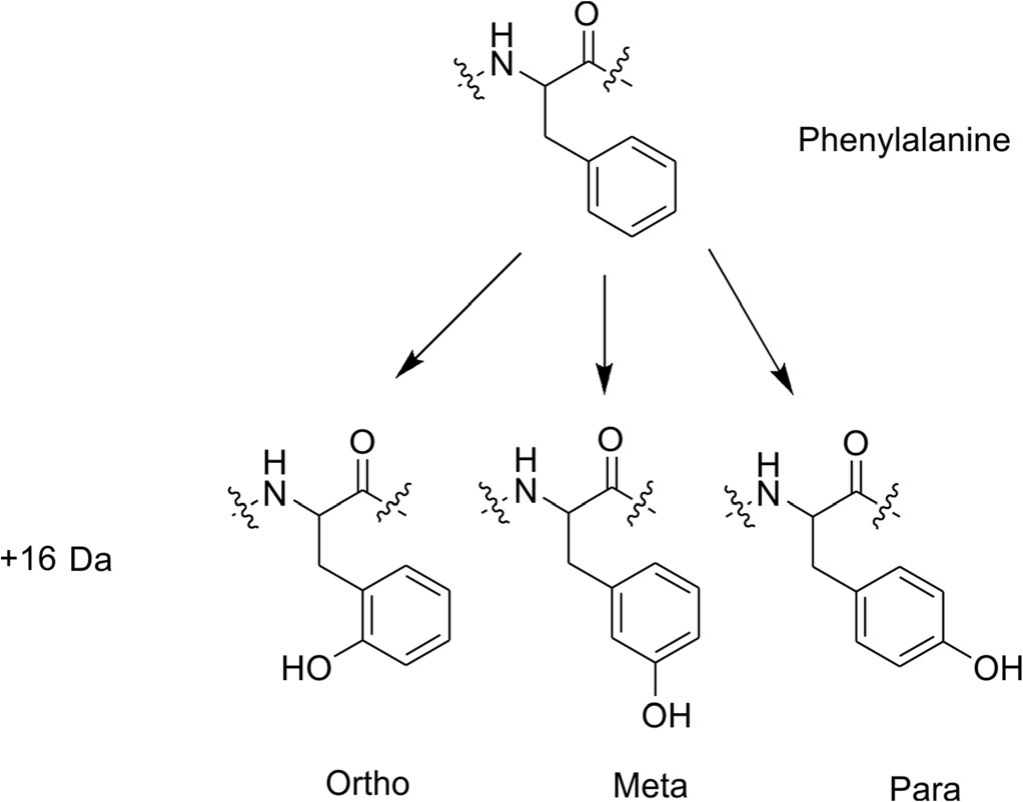
Structural isomers arising from + 16 Da oxidation of phenylalanine

**Scheme 2:**
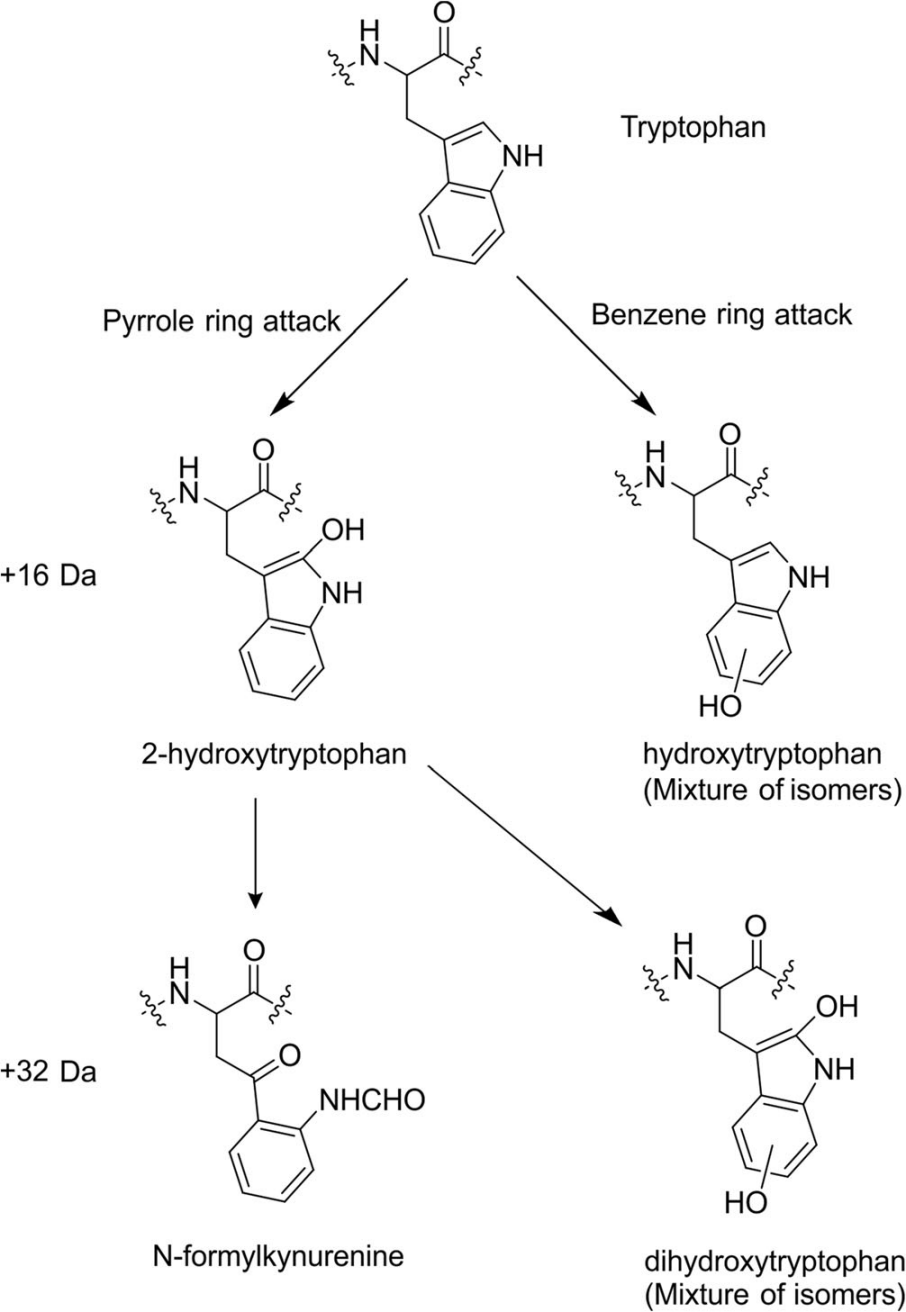
Structural isomers arising from + 16 Da and + 32 Da oxidation of tryptophan

When analysing XICs obtained from FPOP data, each peak separated by reversed phase—UPLC can be assigned a modification site on the peptide from the MS/MS spectrum acquired for a particular *m/z* at a specific retention time. If multiple oxidisable residues are present in the peptide sequence (e.g. His and Lys from within the same chymotryptic peptide) then multiple peaks, at unique retention times, are often observed in the XIC as different, but isobaric, modified species where the peptide has been modified at different residues. Figure 5a shows the XIC of the unmodified peptide containing residues 11–26 of β_2_m (sequence SRHPAENGKSNFLNCY; black trace) overlaid with the XIC of the same peptide with a single oxidation modification (i.e. + 16 Da) (red trace). When analysing the FPOP data, we noted that the same modified peptide species, i.e. where the modification was present on the same residues, eluted at multiple different retention times. An example of this is shown in Figure 5b–d where CID MS/MS identified Phe22 as the modified residue at three different retention times, Tyr26 at two different retention times, and His13 and Lys19 at a single retention time each. This observation has been eluded to elsewhere as the presence of structural isomers generated by hydroxyl radical addition at different positions on the side chains of aromatic amino acids, and is expected based on known amino acid oxidation chemistry [16, 22, 26, 50, 51]. Examples of the multiple oxidation products of Phe and Trp are shown in Schemes 1 and 2, respectively.

**Figure 6:**
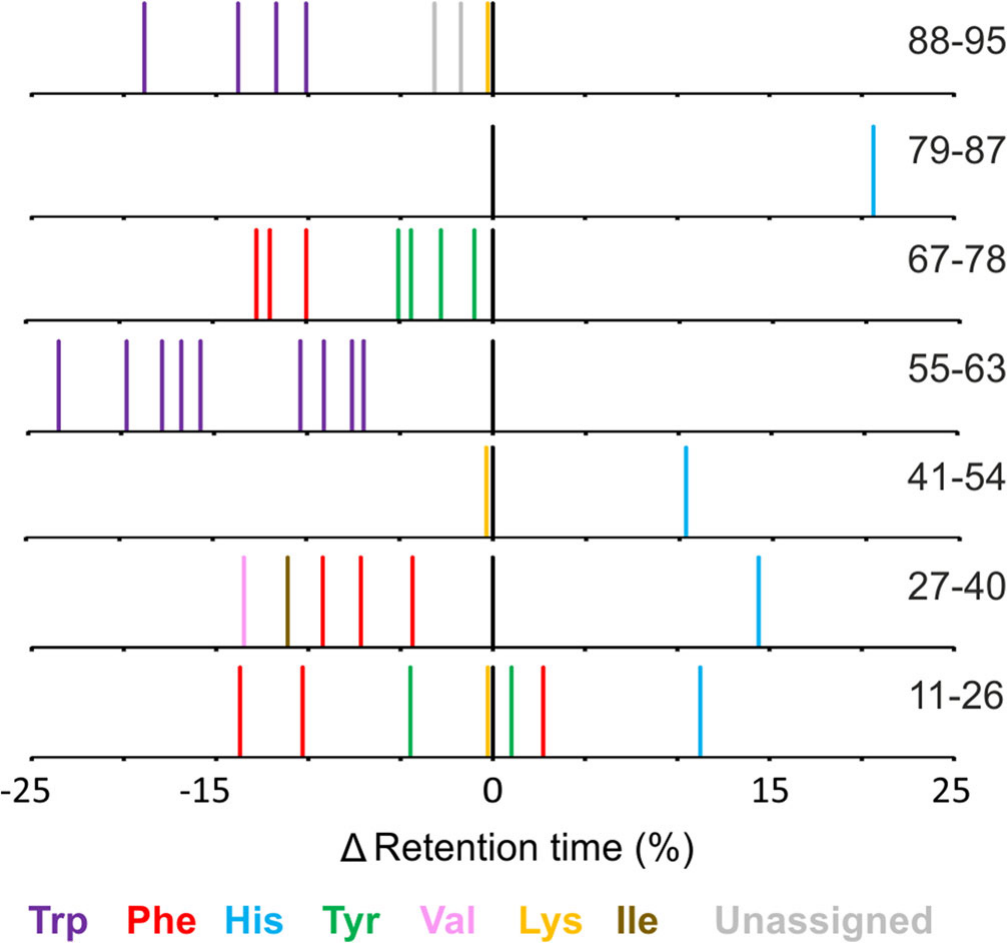
Retention time analysis of FPOP-modified peptide residues. Retention times (x-axis) are shown as a relative difference from the unmodified version of each peptide (black peak at 0) and coloured by the residue assigned to each retention time. Peptide sequences are shown to the right of each trace. Raw XICs for modified and unmodified versions of every peptide not featured in the main text are shown in Supp. Info. Figs. S5-S9

**Figure 7:**
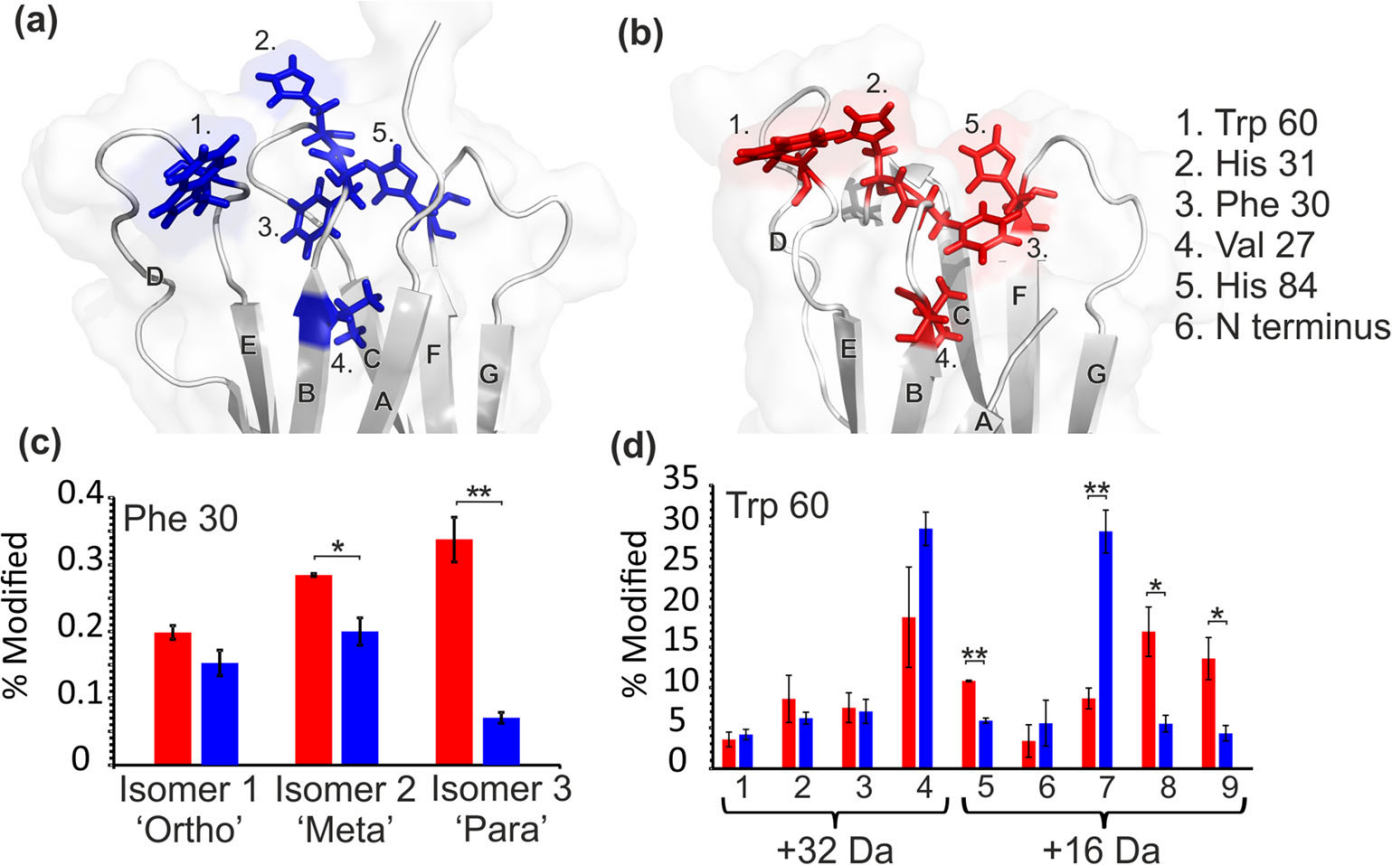
Structures of (**a**) wild-type (PDB: 2XKS) and (**b**) ΔN6 (PDB: 2XKU) β_2_m [29]. The orientation of the side chains of oxidised residues that showed a statistically significant difference in labelling between the two proteins are shown (**a**) in blue (wild-type) and (**b**) in red (ΔN6); residues Val27 (4), His31 (2) and Trp60 (1) show higher oxidative labelling in the wild-type protein, whereas residues Phe30 (3) and His84 (5) underwent more oxidative labelling in ΔN6. β-strands are labelled A-G; the location of the N-terminus with respect to these residues is indicated in (**a**); (**c**) oxidation levels of the three identified Phe30 oxidised species in wild-type β_2_m (blue) and ΔN6 (red). (**d**) Oxidation levels of the nine identified Trp60 species for both + 16 Da and + 32 Da mass additions in wild-type (blue) and ΔN6 (red). X-axis numbering is consistent with XIC peak numbering in Figure 8. * *p* < 0.05, ** *p* < 0.01

As noted in other studies, the presence of multiple structural isomers complicates FPOP data analyses and can obstruct reliable assignment of modified residues [26]. Indeed, data complexity is such that many FPOP studies quantify modifications to the peptide level [17, 26] or, where reliable assignments can be made, sum positional isomers peaks in the XIC to attain residue level information [22]. However, this added complexity becomes apparent upon examination of the retention time data of the modified peptides of both wild-type and ΔN6 β_2_m. Figure 6 shows the mean difference in retention time of each modified peak, relative to the unmodified version of the peptide, for each of the seven chymotryptic peptides characterised. Three peaks were observed for each of the three different oxidised Phe residues identified (Phe22, Phe30 and Phe70), consistent with the *ortho*, *meta* and *para* oxidisable positions (Scheme 1) [16]. Each of the three modified tyrosine residues (Tyr26, Tyr67 and Tyr78) could be assigned two peaks, which is consistent with the possible *ortho* and *meta* isomers (Figure 6). Tryptophan oxidation, however, was the most complex (Scheme 2, Figures 6 and 7). A total of 13 peaks were identified as modified tryptophan despite the presence of only two Trp residues in the ΔN6 sequence (Figure 6). Nine peaks (five + 16 Da and four + 32 Da) were identified for oxidised Trp60, which is consistent with the five expected + 16 Da modifications at four different positions on the benzene ring and one on the pyrrole ring (Scheme 2) [16]. Interestingly, only four + 16 Da peaks were identified for Trp95. Published NMR structures of wild-type and ΔN6 β_2_m [29] indicate that this residue is ~ 90% buried, which is likely to have an effect on the labelling of each isomer, and may mean the final positional isomer is too low in abundance to be observed. Alternatively, the XIC for the + 16 Da modified version of this peptide shows unresolved peaks at 23.5 min (Supp. Info. Fig. S5). Despite having a mass consistent with that expected, the MS/MS spectra at this retention time were of low intensity and it is possible the other Trp isomer is in this unresolved region.

It is interesting to note that the vast majority of FPOP oxidations lead to a decrease in retention time compared with the unoxidised peptide, with the exception of the His oxidations which render the peptide more hydrophobic.

### Assigning and Interpreting Structural Isomers Detected by FPOP

We next sought to achieve a more in-depth understanding of the changes in side chain orientation and/or dynamics of wild-type and ΔN6 β_2_m observed by use of HDX and FPOP, and also to probe the extent to which FPOP structural isomers can be interpreted to gain information on side chain positioning in the two proteins. Using RP-UPLC, the retention time order of the three isomers of oxidised phenylalanine has been reported previously as *para < meta < ortho*, both as free amino acids and when present in a peptide chain [52–56]. Assigning the same retention time order to the oxidised Phe peaks observed here, it is possible to extract more detailed structural information from the available data (Figure 7). Phe30 was found to label ~ twofold more in ΔN6 than in wild-type β_2_m at the residue level (Figures 3a and 4). Interestingly, when quantifying the three isomeric peaks of Phe30 separately, the *para* isomer (shortest retention time) shows a > fourfold difference in labelling between wild-type and ΔN6 β_2_m, while the *ortho* position (longest retention time) shows no statistically significant difference (*p* = 0.067; Figure 7c). The region surrounding Phe30 is well-defined in the NMR structures of both wild-type and ΔN6 β_2_m [29], and is of particular importance as this residue is located directly adjacent to Pro32, which undergoes the *cis-trans* isomerisation which is thought to be an essential step for β_2_m aggregation into amyloid [29, 36]. The NMR structure of wild-type β_2_m shows the *para* position pointing towards the top of the E-strand, and is almost completely secluded from solvent by the E-strand and D-E loop (Figure 7a). By comparison, the NMR structure of ΔN6 indicates that the Phe30 *para* position is pointing directly out of the bulk protein into the solvent, in place of the truncated N-terminal six amino acids (Figure 7b). Using all 30 states of the available NMR structures [29], we calculated the average SASA for each isomer position of Phe30 for wild-type and ΔN6 β_2_m. The average difference in SASA between the two proteins was 1.51 Å^2^, 6.98 Å^2^, and 12.16 Å^2^ for the *ortho*, *meta* and *para* positions of Phe30, respectively. This trend is in good agreement with the FPOP data observed for each of the oxidisable positions (Figure 7c) and supports the assignment made for each isomer. From these FPOP data, we calculated *para*:*meta*:*ortho* ratios of 0.35:1.00:0.76 compared to 1.19:1.00:0.70 for Phe30 for wild-type β_2_m and ΔN6, respectively. Neither ratio matches the expected isomer ratio for free Phe oxidation in solution (1.5:1:2 or 2.1:1:2.3 depending on solution conditions [16]). Similarly, the other two oxidised Phe residues identified (Phe22 and Phe70) do not match this expected *para*:*meta*:*ortho* ratio for the free amino acid. As all three of these Phe residues are, at least partially, buried in the NMR structures of both the wild-type protein and ΔN6 (SASA scores are reported in Supp. Info. Table S2), it is reasonable to conclude that the reduced solvent accessibility of the side chain affects the oxidised isomer distribution, as well as the overall % modified of the residue.

The rotation of the Phe30 side chain in ΔN6 may also offer an explanation of the FPOP behaviour of Val27. All the significantly different FPOP labelled side chains in the region surrounding the N-terminal truncation showed FPOP labelling trends which correlated with the changes observed in calculated SASA from the NMR structures. His84 showed increased labelling in ΔN6 (Figures 3a and 4) and an increase in SASA of ~ 28%. Trp60 and His31 both showed decreased labelling in ΔN6, and, on average, had lower SASAs in the ΔN6 NMR structure (~ 38% and ~ 4% decrease respectively). Val27, however, showed ~ threefold lower FPOP labelling in ΔN6 (Figures 3a and 4), despite a ~ 22-fold higher average SASA. A possible explanation for this is the increased competition for hydroxyl radicals caused by the solvent exposure of the nearby highly reactive Phe30 side chain in ΔN6. Although sequence effects of FPOP labelling have not been well characterised, thermal unfolding experiments have shown that poorly reactive residues undergo less FPOP labelling in unfolded proteins due to increased competition from solvent-exposed reactive side chains [49].

Trp60, which at the residue level showed a small but statistically significant decrease in oxidative labelling in ΔN6 compared with wild-type β_2_m (Figure 3a), reveals a more complex picture when quantified to the isomer level. The D-E loop region surrounding Trp60 is ill-defined in the NMR structures of both proteins, suggestive of a dynamic nature and making a detailed structural comparison with the FPOP data challenging. The complexity of these data is such that isomer assignments of this residue could not be made with certainty. However, careful examination of the data permits some tentative assignments to be made.

**Figure 8:**
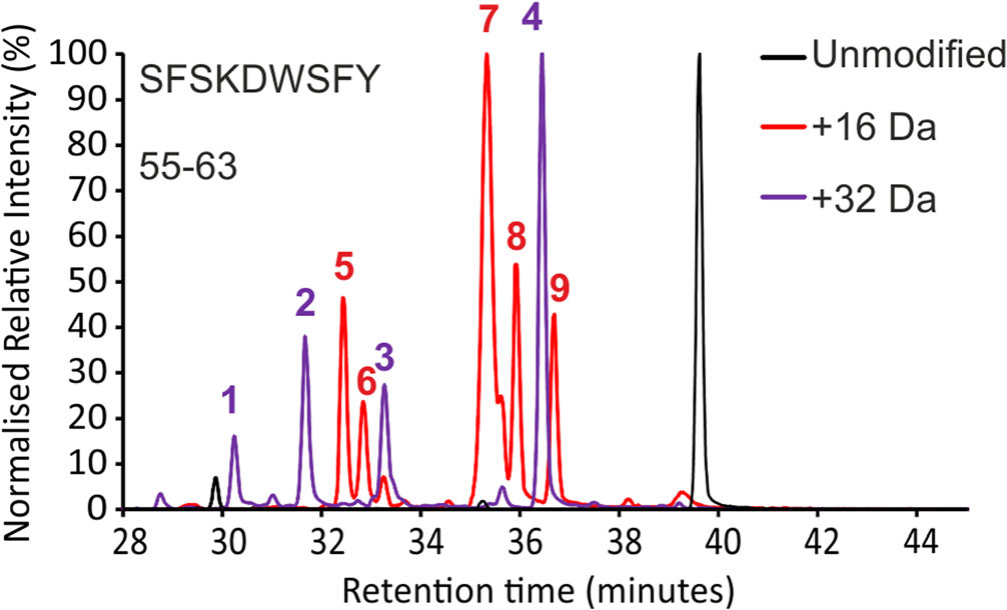
FPOP modifications of peptide 55–63 (sequence SFSKDWSFY). XIC of unmodified (black), + 16 Da modified (red) and + 32 Da modified (purple) peptide. Peaks 1–9 were identified as different oxidised Trp60 products. Quantification of peaks 1–9 is shown in Figure 7d)

Hydroxyl radicals are reported to attack the pyrrole moiety of the Trp side chain preferentially with a ratio of 60:40 compared to the adjoining benzene ring [16, 57]. The two major products of pyrrole ring oxidation and subsequent ring opening are 2-hydroxytryptophan (+ 16 Da) and N-formylkynurenine (+ 32 Da), which are consistent with the two most intense-modified peaks in wild-type β_2_m (peaks 7 and 4, respectively, in Figures 7d and 8) each accounting for *ca* 30% of the total oxidation on Trp60. Indeed, comparing peaks 7 and 4 to the other Trp60 modifications in the wild-type protein gives a ratio of 60.4:39.6 in favour of the dominant + 16 Da and + 32 Da modifications. This is in good agreement with the expected modification ratio of free Trp in solution [16, 57], and can be rationalised from the NMR structure of the wild-type protein which shows Trp60 as ~ 80% solvent exposed. Additionally, peak 7, the major + 16 Da modified peak for the wild-type protein tentatively assigned as the 2-hydroxytryptophan product, has a smaller shoulder peak at approximately 30% intensity relative to the major peak (Figure 8). 2-hydroxytryptophan is known to exist as two diastereomers [58]. These diastereomers interconvert during chromatography by keto-enol tautomerism and are observed as a double peak in RP-UPLC [59]. This could account for the observation of the smaller shoulder peak of this product in the XIC.

In the case of ΔN6, peaks 7 and 4 (Figures 7d and 8) both have lower average % modified values, and for peak 7 (+ 16 Da) this is greater than a threefold change. Other covalent labelling MS methods have shown burial of Trp60 in other amyloidogenic variants of β_2_m relative to the wild-type protein [60]. Similarly, partial proteolysis experiments have revealed that Trp60 in ΔN6 is less accessible to proteases [34]. However, three of the four remaining + 16 Da Trp60 modifications show the opposite trend, with significantly *more* labelling in ΔN6 relative to the wild-type protein (peaks 9, 8 and 5, Figures 7d and 8). Assuming the previous assignments are correct, these remaining + 16 Da peaks would likely correspond to the four isomers of hydroxytryptophan resulting from benzene ring oxidation (Scheme 2). Combined, these data suggest a rearrangement of the side chain, rather than complete burial where, on average, the pyrrole ring becomes less solvent exposed in ΔN6, and the benzene ring more so. The three remaining + 32 Da peaks (peaks 1–3, Figures 7d and 8) are likely isomers of dihydroxytryptophan, resulting from both pyrrole and benzene ring oxidation [16].

Together, these results illustrate that FPOP is capable of distinguishing differences in solvent accessibility down the sub-amino acid level, and that the labelling trends of such isomers can be interpreted meaningfully to gain higher resolution information on protein structure.

## Conclusions

HDX and FPOP are both powerful techniques for the study and characterisation of protein structure and dynamics. While peptide level HDX allows characterisation of changes in backbone dynamics and hydrogen bonding, typically on greater than millisecond timescales, with broad but low resolution coverage of a protein’s sequence, FPOP is capable of comparing the solvent accessibility of individual residues on much shorter (μs-ms) timescales.

The FPOP data acquired here demonstrate how oxidative labelling of a protein, followed by proteolysis and LC-MS/MS analysis, can lead to the identification of structural isomers associated with oxidation of aromatic amino acid residues. Using affirmed orders of elution of these isomers, their relative abundance can be interpreted to help describe the side chain orientation of such hydrophobic residues within a protein structure, as illustrated by the range of Phe30 structural isomers detected for both wild-type β_2_m and ΔN6. These side chain rearrangements are undetectable by HDX experiments and further illustrate the complementary nature of the two methods.

While the appearance of structural isomers in FPOP complicates the data analysis significantly, it also provides an exciting opportunity to obtain unique information once methods are developed for the routine characterisation of these modifications in the FPOP workflow. The oxidation products of both Phe [61] and Trp [62] are distinguishable by differential UV absorbance spectra. UV detection is routinely implemented into LC-MS workflows. Indeed, online UV absorbance coupled with mass spectrometry has been used previously to characterise unwanted Trp oxidation as degradation products of heat stressed antibodies [63]. This methodology could be employed to confirm the tentative assignments made for Trp60 in this study. Although routine analysis of structural isomers by LC may require long gradients to enable high quality LC separation of peptides, long LC gradients are no barrier in FPOP analysis due to the covalent and irreversible nature of the labelling. Additionally, progress by others has already been made in implementing multi-dimensional LC to improve separation and identification of FPOP peptides [64]. That said, additional studies on other well-characterised systems would be necessary to assess the usefulness of interpreting positional isomers in FPOP. Similarly, detailed data interpretation of structural isomers, and indeed residue level FPOP data, would benefit greatly from a deeper understanding of the effect of nearby side chains, the amino acid sequence and solution conditions on FPOP labelling, and the influence these factors have on the relationship between SASA and the degree of modification.

In terms of the amyloidogenic proteins under scrutiny here, protein aggregation is frequently dependent on a partial unfolding event involving surface exposure of hydrophobic residues [27]. These often subtle structural changes can be difficult to probe by other structural biology methods depending on the amplitude and timescale of the motions involved. As a technique particularly sensitive to labelling hydrophobic residues, and doing so in explicit detail, sub-amino acid level FPOP could prove invaluable to the study of protein assembly mechanisms. Moreover, the potential for detailed FPOP mapping of hydrophobic side chain orientations within aggregation-prone regions offers the promise of using FPOP to aid the design and selection of small molecule inhibitors, in addition to other applications such as the analysis protein-protein and protein-ligand interactions in large and dynamic protein assemblies.

## Electronic Supplementary Material

ESM 1 (PDF 884 kb)
