# Supplementary material for: Comparing Hydrogen Deuterium Exchange and Fast Photochemical Oxidation of Proteins: a Structural Characterisation of Wild-Type and ΔN6 β2-Microglobulin
Source: J Am Soc Mass Spectrom. 2018 Sep 28;29(12):2413–26. doi: 10.1007/s13361-018-2067-y (PMC6276068; doi:10.1007/s13361-018-2067-y)
Supplement: Supplementary file 1 — (PDF 884 kb) [file 13361_2018_2067_MOESM1_ESM.pdf]

Comparing Hydrogen Deuterium Exchange and Fast Photochemical Oxidation of Proteins: a structural characterisation of wild-type β_2-microglobulin and ΔN6

Owen Cornwell, Sheena E. Radford, Alison E. Ashcroft, James R. Ault

Astbury Centre for Structural Molecular Biology, Faculty of Biological Sciences, University of Leeds, Leeds, LS2 9JT, UK

SUPPLEMENTARY INFORMATION

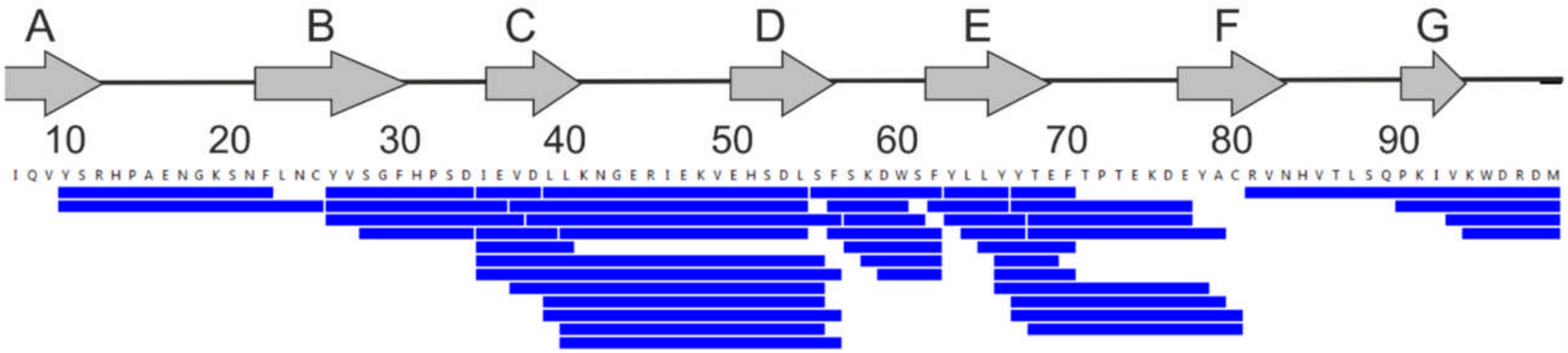

Figure S1. Peptide coverage of β_2m from the HDX-MS experiment (considering only the residues from 7-100 in order to compare wild-type β_2m and ΔN6).

Peptides shown are those that passed the identification criteria (see Methods section in the Main Text for details) in *both* proteins so that a like-for-like comparison of uptake data could be made for the whole peptide list. Residues are numbered as in the full sequence for recombinant wild-type β_2m; β-strands are labelled A – G (PDB: 2XKS).

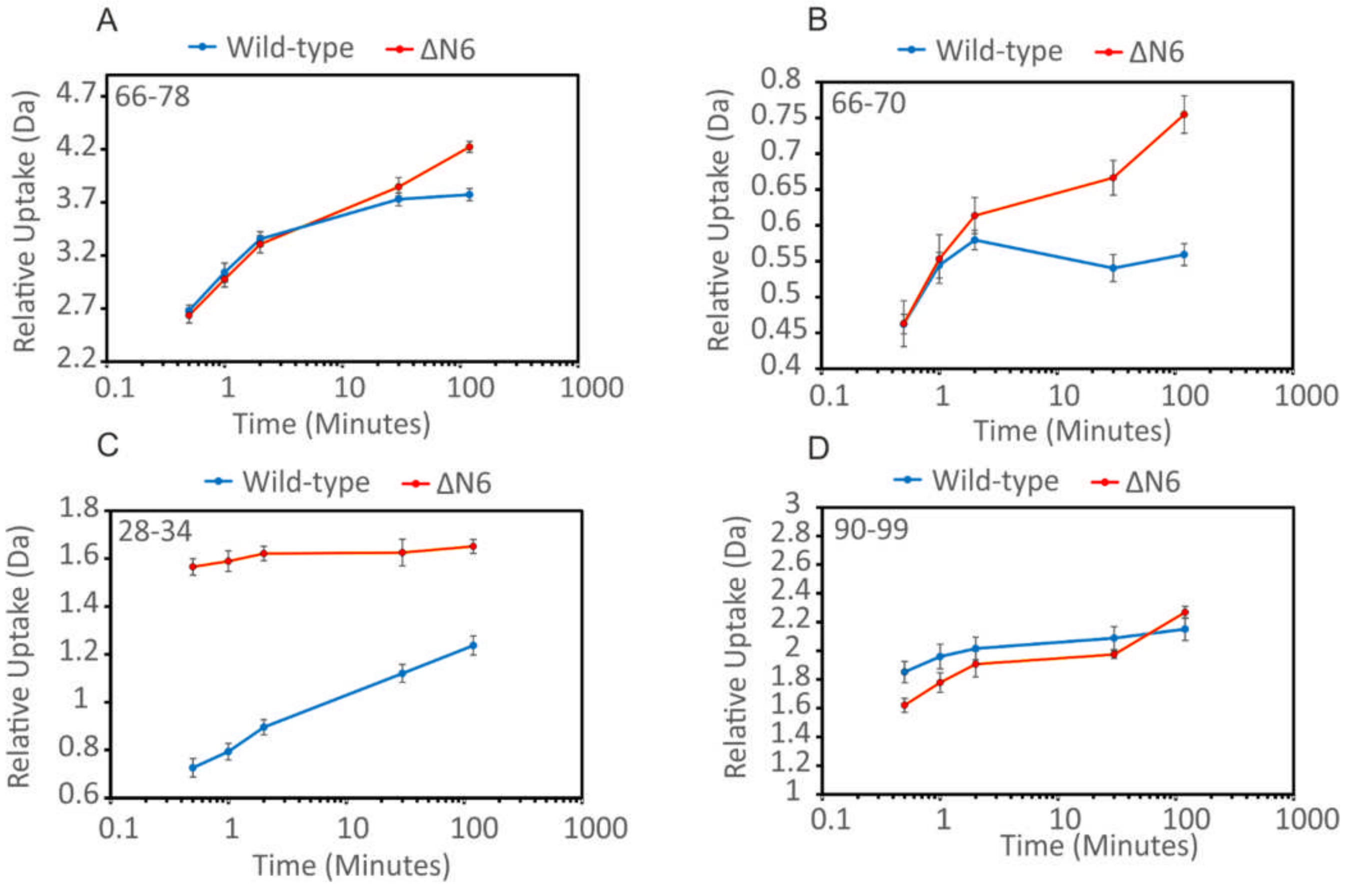

Figure S2. HDX uptake plots of wild-type (blue) and ΔN6 (red) β_2m showing regions of difference between wild-type β_2m and ΔN6.

These peptides overlap with the ones highlighted in the Main Text, Fig. 2. The residues covered by each peptide are shown in the top left corner of each plot.

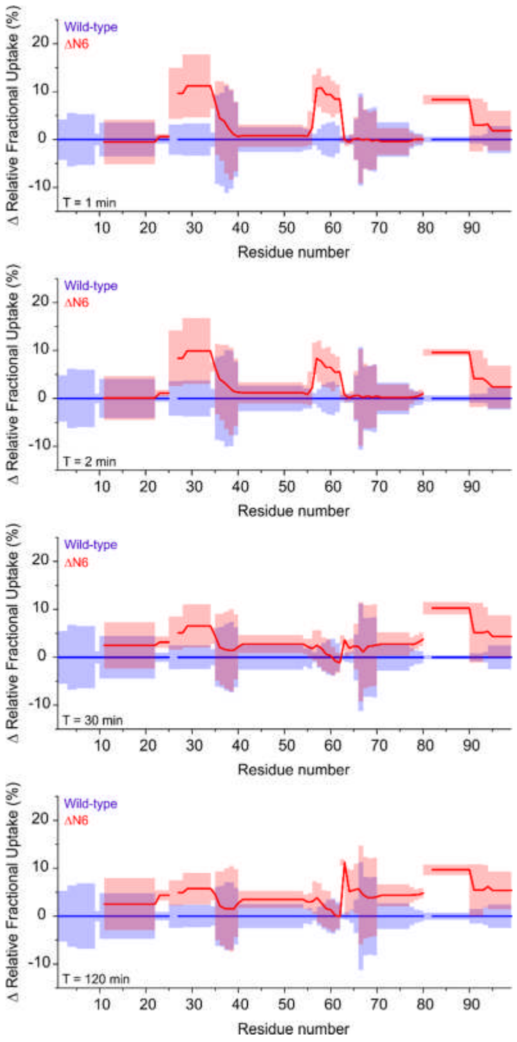

Figure S3. PAVED difference plots for wild-type β_2m and ΔN6 HDX-MS data.

Shaded regions show combined standard deviation per residue for wild-type (blue) and ΔN6 (red). Time-points (1 min, 2 min, 30 min and 120 min) are shown in the bottom left corner of each difference plot.

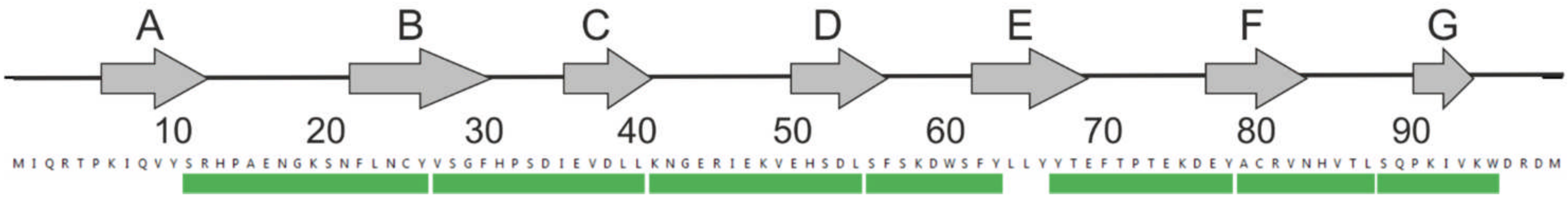

Figure S4. Peptide coverage of β_2m from the FPOP quantification experiment (considering only the residues from 7-100 in order to compare wild-type β_2m and ΔN6).

Peptides (green bars) shown are those that were identified reproducibly using LC-MS/MS in both wild-type β_2m and ΔN6 after FPOP photolysis followed by chymotrypsin digestion. Residues are numbered as in the full sequence for recombinant wild-type β_2m. The β-strands are labelled A – G.

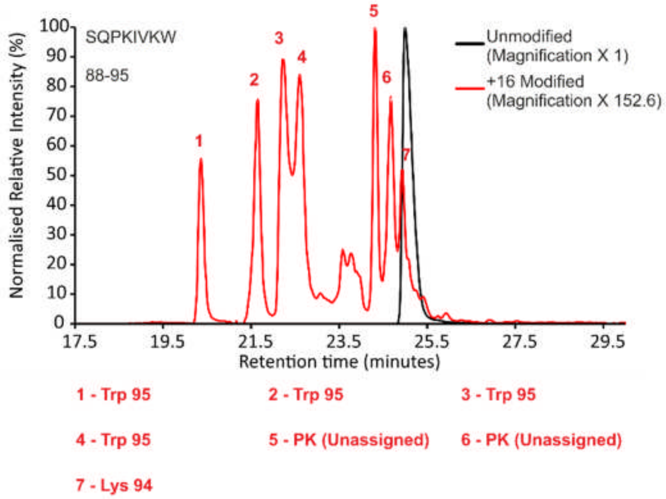

Figure S5. Extracted Ion Chromatograms (XIC) from LC-MS/MS data showing FPOP oxidations for peptide 88-95 (sequence SQPKIVKW).

Overlaid extracted ion chromatograms of the unmodified peptide (black peak) and +16 Da modified peptides (red peaks).

Table S1. Summary of raw data and interpretation for the identified FPOP modifications.

Unmodified		Modified						
**Peptide**	***m/z***	**Δmass (Da)**	***m/z***	**ΔRT (± S.D) (min)**	**Wild-type (% Modified ± S.D)**	**ΔN6 (% Modified ±S.D)**	**Assignment**	**Assignment Criteria**
**SRHPAENGKSNFLNCY (11-26)**	947.43 (2+)<br>631.96 (3+)<br>474.22 (4+)	+16	478.21 (3+)	+2.67 ± 0.08	1.41 ± 0.29	1.89 ± 0.45	His 13	MS/MS
		+16	478.21 (4+)<br>637.28 (3+)	+0.65 ± 0.04	0.23 ± 0.04	0.23 ± 0.02	Phe 22	MS/MS
		+16	478.21 (4+)<br>637.28 (3+)	+0.24 ± 0.07	0.21 ± 0.04	0.18 ±0.05	Tyr 26	MS/MS
		+16	478.21 (4+)<br>637.28 (3+)	-0.064 ± 0.04	0.51 ± 0.04	0.56 ± 0.04	Lys 19	MS/MS
		+16	478.21 (4+)<br>637.28 (3+)	-1.06 ± 0.06	0.47 ± 0.08	0.66 ± 0.05	Tyr 26	MS/MS
		+16	478.21 (4+)<br>637.28 (3+)	-2.46 ± 0.07	0.18 ± 0.00	0.24 ± 0.04	Phe 22	MS/MS
		+16	478.21 (4+)<br>637.28 (3+)	-3.27 ± 0.06	0.21 ± 0.02	0.52 ± 0.14	Phe 22	MS/MS
**VSGFHPSDIEVDLL (27-40)**	764.38 (2+)	+ 16	772.38 (2+)	+6.55 ± 0.26	0.39 ± 0.04	0.26 ± 0.02	His 31	MS/MS
		+16	772.38 (2+)	-1.98 ± 0.18	0.15 ± 0.02	0.20 ± 0.01	Phe 30	MS/MS
		+16	772.38 (2+)	-3.26 ± 0.28	0.20 ± 0.02	0.28 ± 0.00	Phe 30	1. MS/MS narrows modification site to GFH<br>2. Phe is >405X more reactive than Gly<br>3. Although His and Phe have similar reactivities – His on this peptide was already assigned and has only ever observed here as one +16 Da peak<br>4. All other +16 Da His modifications identified have retention times *longer* than the unmodified peak

		+16	772.38 (2+)	-4.20 ± 0.29	0.07 ± 0.01	0.34 ± 0.03	Phe 30	MS/MS
		+16	772.38 (2+)	-5.06 ± 0.46	0.05 ± 0.01	Not detected	Ile 35	MS/MS
		+16	772.38 (2+)	-6.14 ± 0.32	0.13 ± 0.01	0.06 ± 0.02	Val 27	MS/MS
**KNGERIEKVEHSDL (41-54)**	827.43 (2+)<br>551.96 (3+)<br>414.22 (4)	+16	557.29 (3+)	+1.86 ± 0.05	0.96 ± 0.62	2.27 ± 0.23	His 51	MS/MS
		+16	557.29 (3+)	- 0.06 ± 0.04	0.19 ± 0.05	0.17 ± 0.11	Lys 48	MS/MS
**SFSKDWSFY (55-63)**	583.76 (2+)	+16	591.76 (2+)	-2.74 ± 0.05	4.32 ± 0.95	13.58 ± 2.63	Trp 60	MS/MS
		+16	591.76 (2+)	-3.58 ± 0.15	5.51 ± 1.02	16.93 ± 3.05	Trp 60	MS/MS
		+16	591.76 (2+)	-4.08 ± 0.11	29.31 ± 2.63	8.65 ± 1.29	Trp 60	MS/MS
		+16	591.76 (2+)	-6.61 ± 0.07	5.57 ± 2.83	3.36 ± 2.01	Trp 60	MS/MS
		+16	591.76 (2+)	-7.02 ± 0.06	5.90 ± 0.32	10.82 ± 0.09	Trp 60	MS/MS
		+32	599.75 (2+)	-2.99 ± 0.05	29.65 ± 2.04	18.71 ± 6.23	Trp 60	MS/MS
		+32	599.75 (2+)	-6.20 ± 0.05	7.03 ± 1.48	7.48 ± 1.85	Trp 60	MS/MS
		+32	599.75 (2+)	-7.77 ± 0.08	6.20 ± 0.75	8.58 ± 2.92	Trp 60	MS/MS
		+32	599.75 (2+)	-9.21 ± 0.09	4.16 ± 0.64	3.55 ± 0.91	Trp 60	1. MS/MS identifies modification site to WSFY<br>2. Trp is the most reactive residue on the peptide<br>3. All 8 other modified peaks in the XIC were Trp 60
**YTEFTPTEKDEY (67-78)**	761.83 (2+)	+16	769.82 (2+)	-0.26 ± 0.04	0.35 ± 0.01	0.30 ± 0.07	Tyr 67	1. MS/MS narrows modification site to YTE<br>2. Tyr is >25X more reactive than Thr and > 56X more reactive than Glu
		+16	769.82 (2+)	-0.74 ± 0.04	0.78 ± 0.13	0.94 ± 0.02	Tyr 78	MS/MS
		+16	769.82 (2+)	-1.16 ± 0.04	0.06 ± 0.01	0.06 ± 0.01	Tyr 67	1. MS/MS narrows modification site to YT<br>2. Tyr is 25X more reactive than Thr
		+16	769.82 (2+)	-1.34 ± 0.05	0.09 ± 0.01	0.08 ± 0.01	Tyr 78	1. MS/MS narrows modification site to KDEY<br>2. Tyr is >34X more reactive than Lys, 56X more reactive than Glu and >173X more reactive than Asp

		+16	769.82 (2+)	-2.65 ± 0.06	0.02 ± 0.00	0.02 ± 0.00	Phe 70	MS/MS
		+16	769.82 (2+)	-3.17 ± 0.06	0.03 ± 0.00	0.03 ± 0.00	Phe 70	MS/MS
		+16	769.82 (2+)	-3.36 ± 0.07	0.02 ± 0.00	0.02 ± 0.00	Phe 70	MS/MS
**ACRVNHVTL (79-87)**	535.28 (2+)<br>357.19 (3+)	+16	362.52 (3+)	+4.37 ± 0.07	0.12 ± 0.03	0.45 ± 0.07	His 84	MS/MS
**SQPKIVKW (88-95)**	493.29 (2+)	+16	501.29 (2+)	-0.07 ± 0.07	0.18 ± 0.01	0.21 ± 0.01	Lys 94	MS/MS
		+16	501.29 (2+)	-0.44 ± 0.12	0.39 ± 0.01	0.32 ± 0.03	Unassigned	1. MS/MS narrows modification to Pro 90 or Lys 91<br>2. Both have similar reactivities (approx. 2 fold difference)<br>3. Pro can have >1 structural isomer
		+16	501.29 (2+)	-0.79 ± 0.11	0.35 ± 0.03	0.32 ± 0.01	Unassigned	1. MS/MS narrows modification to Pro 90 or Lys 91<br>2. Both have similar reactivities (approx. 2 fold difference)<br>3. Pro can have >1 structural isomer
		+16	501.29 (2+)	-2.54 ± 0.14	0.35 ± 0.01	0.35 ± 0.11	Trp 95	MS/MS
		+16	501.29 (2+)	-2.94 ± 0.14	0.43 ± 0.07	0.39 ± 0.08	Trp 95	MS/MS
		+16	501.29 (2+)	-3.46 ± 0.14	0.17 ± 0.07	0.36 ± 0.02	Trp 95	MS/MS
		+16	501.29 (2+)	-4.73 ± 0.13	0.15 ± 0.08	0.16 ± 0.04	Trp 95	MS/MS

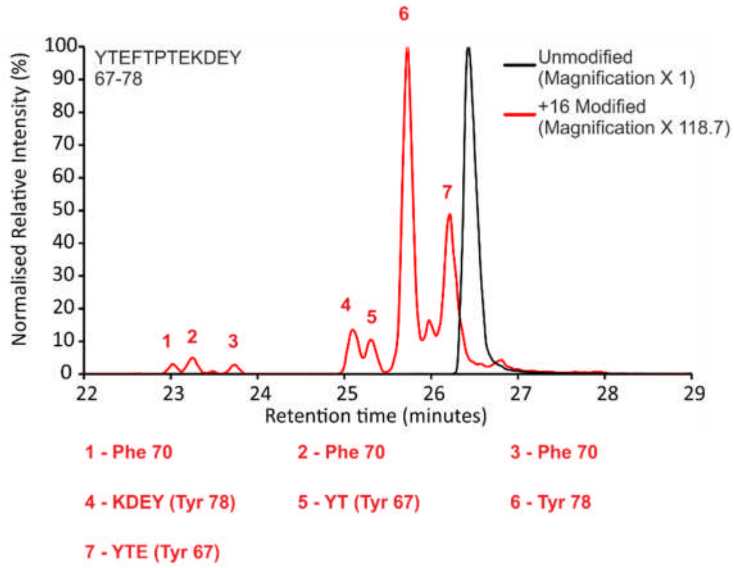

Figure S6. Extracted Ion Chromatograms (XIC) from LC-MS/MS data showing FPOP oxidations for peptide 67-78 (sequence YTEFTPTEKDEY).

Overlaid extracted ion chromatograms of the unmodified peptide (black peak)and +16 Da modified peptides (red peaks).

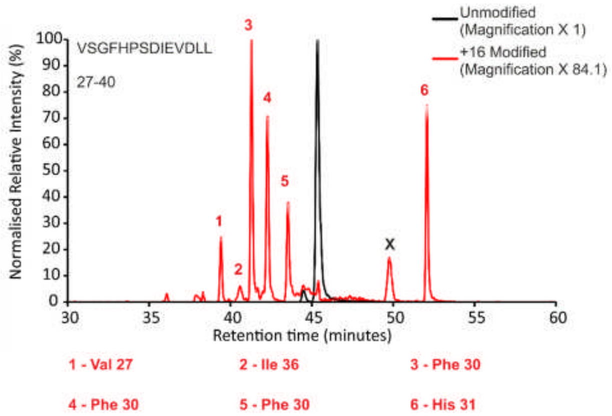

Figure S7. Extracted Ion Chromatograms (XIC) from LC-MS/MS data showing FPOP oxidations for peptide 27-40 (sequence VSGFHPSDIEVDLL).

Overlaid extracted ion chromatograms of the unmodified peptide (black peak) and +16 Da modified peptides (red peaks). Black X indicates a peptide not identified as present in the sequence of either wild-type or ΔN6 β_2m.

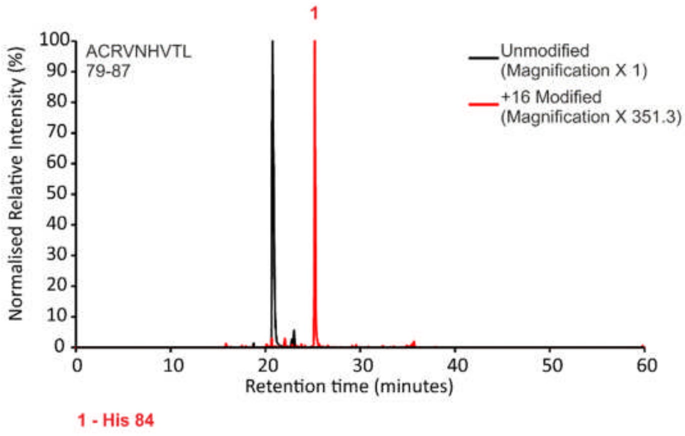

Figure S8: Extracted Ion Chromatograms (XIC) from LC-MS/MS data showing FPOP oxidations for peptide 79-87 (sequence ACRVNHVTL).

Overlaid extracted ion chromatograms of the unmodified peptide (black peak)and +16 Da modified peptides (red peaks).

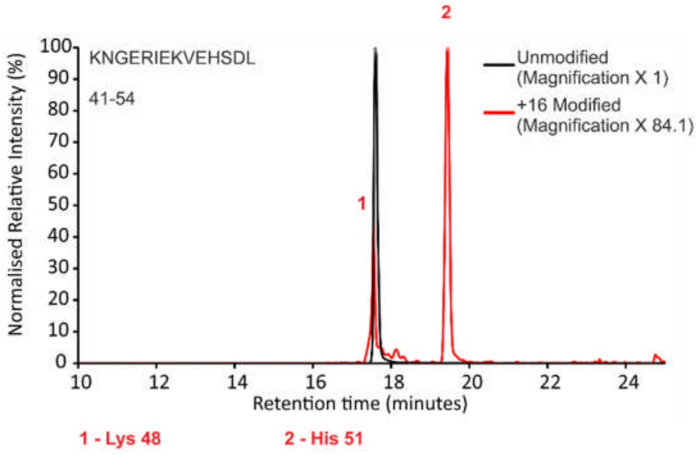

Figure S9. Extracted Ion Chromatograms (XIC) from LC-MS/MS data showing FPOP oxidations for peptide 41-54 (sequence KNGERIEKVEHSDL).

Overlaid extracted ion chromatograms of the unmodified peptide (black peak)and +16 Da modified peptides (red peaks).

Table S2: ***para : meta : ortho*** **ratios of each of the phenylalanine residues identified by FPOP in wild-type and ΔN6 β_2m.**

Residue	Phe22		Phe30		Phe70	
Protein	Wild-type	ΔN6	Wild-type	ΔN6	Wild-type	ΔN6
Isomer Ratio (P:M:O)	1.16:1:1.27	2.15:1:0.94	0.35:1:0.76	1.19:1:0.70	0.57:1:0.54	0.47:1:0.52
SASA (Å^2)	~88.3	~105.2	~2.2	~48.8	~11.0	~5.7
% solvent exposed	~49%	~58.4%	~1.2%	~27.1%	~6.1%	~3.2%
